# Comprehensive temperature controller with internet connectivity for plant growth experiments

**DOI:** 10.1016/j.ohx.2021.e00238

**Published:** 2021-10-14

**Authors:** Kyle McDowell, Yang Zhong, Kira Webster, Hector Jaime Gonzalez, A Zachary Trimble, Camilo Mora

**Affiliations:** a Department of Electrical Engineering, University of Hawaii, 2500 Campus Rd, Honolulu, HI 96822, USA; b Department of Geography, University of Hawaii, 2500 Campus Rd, Honolulu, HI 96822, USA; c Department of Electrical Engineering, Universidad del Valle, Colombia, Valle, Colombia, USA; d Department of Mechanical Engineering, University of Hawaii, 2500 Campus Rd, Honolulu, HI 96822, USA

**Keywords:** Plants, Automation, Temperature control, Temperature monitoring, Open source

## Abstract

The experimental control of temperature is key to many scientific and applied endeavors, particularly for studying the effects of greenhouse-gas driven warming on plant performance. Unfortunately, numerous nuisances in the control of temperature for plants renders most commercially available controllers unsuitable or too expensive. Here we describe a simple to use but comprehensive temperature controller for plant growth experiments in enclosed spaces like nurseries or chambers. The device uses Pulse Width Modulation to control independent cooling and heating elements over a broad range of amperages, which minimizes or eliminates temperature overshoot and ensures precise and accurate temperature control (i.e., sensor accuracy = 0.1 °C; controller accuracy = 0.3 °C.). The device incorporates an internal clock for controlling temperature (and growth lights) concurrent with diurnal cycles, and it has an integrated Wi-Fi chip to transfer sensor data to a web-page, where data are displayed in real time. The device uses off-the-shelf parts and can be built for around $USD63. The controller can be integrated with other reported controllers (e.g., soil moisture and CO2) to produce an affordable multi-system controller necessary for complex factorial experiments, which hopefully can help to accelerate our understanding about the impacts of climatic variables on plant performance.

## Graphical abstract

**Figure:**
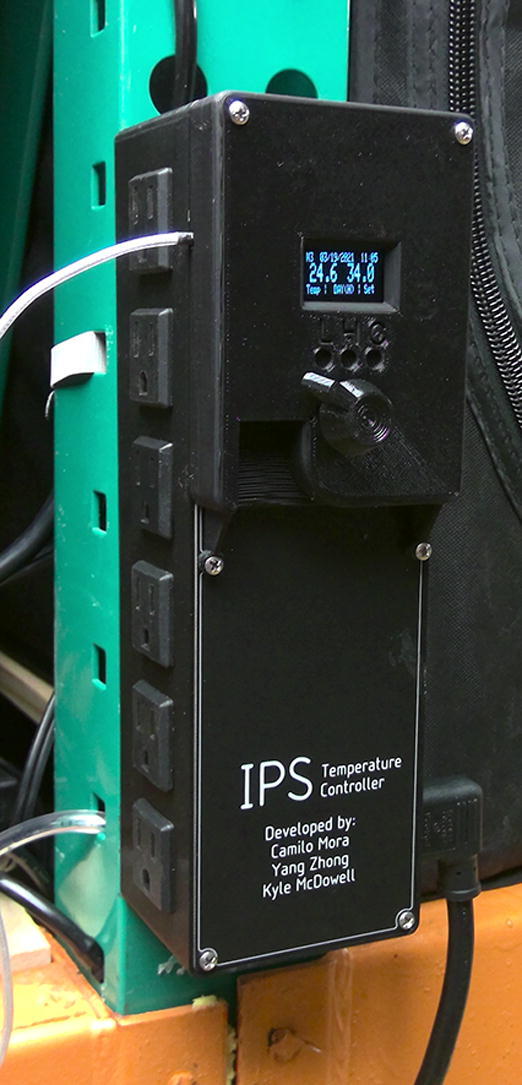

Specifications table

**Table:** 

Hardware name	Comprehensive temperature controller for plant growth experiments
Subject area	• Engineering and Material Science • Environmental, Planetary and Agricultural Sciences • Educational Tools and Open Source Alternatives to Existing Infrastructure
Hardware type	• Measuring and controlling physical properties and in-lab sensors and controls • Field measurements, sensors and controls • Electrical engineering
Open Source License	*CC BY 4.0*
Cost of Hardware	*$USD63*
Source File Repository	https://doi.org/10.17605/OSF.IO/J7ZKV

## 1 Hardware in context

Temperature is a fundamental physical variable for biological processes ranging from the cellular to the individual level and beyond. Not surprisingly the monitoring and control of temperature is central to many applied and research endeavors, with a diversity of commercial solutions readily available for the purpose of monitoring and controlling temperature. The control of temperature is simple in principle: a microcontroller gathers data from a temperature sensor and activates an output channel, in which a heating or cooling element is connected, until a set threshold is reached. However, for certain applications the control of temperature includes several nuisances that make commercially available controls unsuitable or too expensive. In this specific paper, we introduce a temperature controller for the purpose of controlling temperature in enclosed spaces (e.g., plant nurseries or chambers) to study plant growth. For this application, commercially available temperature controllers have specific characteristics that restrict their use:

First, the accuracy with which temperature is measured. For plant experiments, temperature commonly requires accurate measurements, ideally below 0.5 °C as to prevent large phenotypic variation, which can translate into statistical noise when performing analyses.

Second, the need to avoid temperature overshoot (i.e., temperature inertia leading to temperature significantly exceeding the set threshold) and hysteresis (i.e., large temperature oscillations around the set temperature). Typically, controllers turn off the heating or cooling element when the set point temperature is reached; this leads to temperature surpassing the set point by a magnitude determined by the heating or cooling potential of the element, the thermal inertia of the system, and the thermodynamic and heat transfer properties of the system. Lags in sensing temperature and compensating for heat loses add to hysteresis oscillations. A large overshoot can be damaging, at times lethal, to plants, whereas hysteresis adds to noise in plant responses. A key approach to reduce overshoot and hysteresis is Pulse Width Modulation (PWM). PWM controls the time lapses in which heating or cooling elements are On and Off; fine-tuning the PWM On and Off timings allows for heat dissipation and homogenization reducing the magnitude of temperature oscillations around the set threshold. PWM functionally is rarely available in consumer-based controllers.

Third, the ability to control the output channels for the heating and cooling elements with independent PWMs. As mentioned earlier, a common approach to control for overshoot and hysteresis is PWM. However, the fast alternation between power On and Off may be deleterious to some cooling elements. Heating elements, like electrical resistances and fans, can handle rapid On-Off cycling, but some cooling elements, like ACs that utilize compressors, are prone to damage with fast On-Off cycling. Incorporating different PWM cycles for the different output channels will reduce risk to the elements. A somewhat related issue is the need for output channels rated at large amperages to activate large heating or cooling elements. On most available controls, additional Solid-State-Relays are commonly needed to activate high amperage elements.

Fourth, the capability for simultaneous heating and cooling. Most commercially available controls include one output channel, which activates a heater if the device is set to heat or a cooler if the device is set cool. For plant experiments, having the simulations capacity to heat and cool can allow for the control of temperature within more precise and accurate ranges, while facilitating to run experiments at temperatures hotter or colder than the ambient environment without additional parts or settings.

Fifth, the need for a time-dependent control of temperature. Plants require a light and dark phase for photosynthesis, which is activated by sunlight or lack thereof. Depending on the season and location in Earth, variations in temperature between day and night can range from a few degrees to 10 °C or more. Most commercial temperature controls lack this functionality, and thus, they have to be integrated in complex electrical circuits that include timers and at least two temperature controllers, as to allow day and night temperatures. A somewhat related limitation is the need for an additional output channel to control grow lights, so that lights can be paired with set temperatures for light and dark phases.

Sixth, the need to store the sensor data. Available high-end temperature controllers include this functionality in the form of memory cards, serial connectors or wireless communication. For most experimental studies, it is commonly required to report the actual level of control of temperature, which requires access to the raw temperature data over the lapse of the given experiment.

The nuisances outlined above are specific to the control of temperature for plant grow experiments in enclosed chambers or nurseries, but may be common to other applications. Numerous commercially available temperature controls include specifications to deal with one or more of these nuisances, but the extent to which they are all covered commonly correlates to a higher controller price from several hundred dollars to a few thousands [1], [2]. The high price of controllers that address most or all of these nuisances affects the economic feasibility of large-scale experiments, particularly for experiments in which numerous controllers are required.

## 2 Hardware description.

**Fig. 1:**
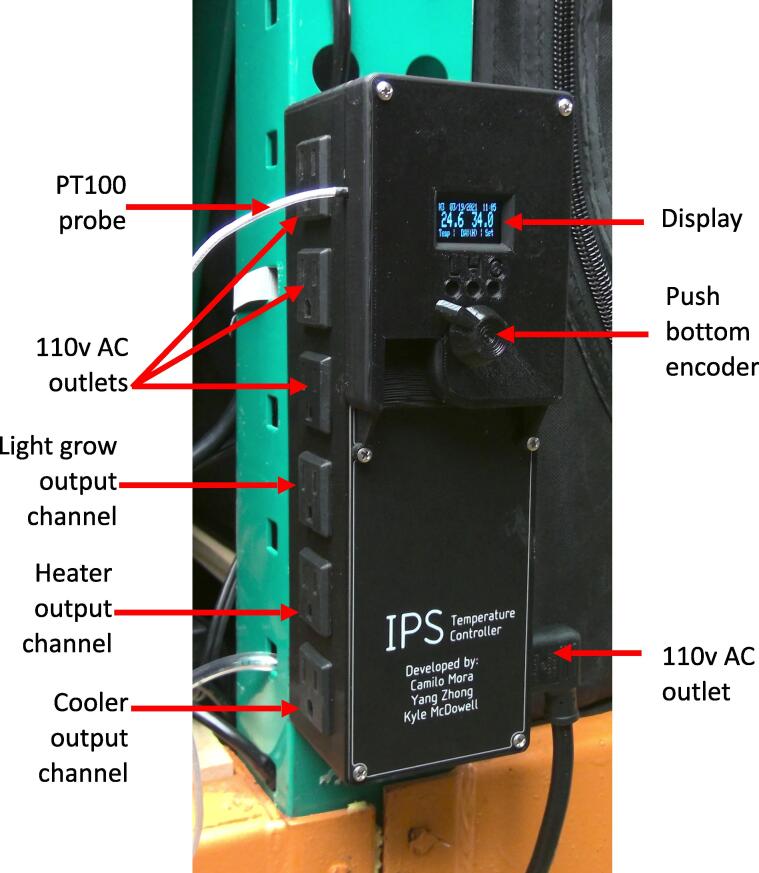
Temperature controller photograph and callout.

Here we report a temperature controller (Fig. 1 that addresses all the nuisances indicated above while remaining affordable. The temperature controller uses a Platinum Resistance Thermometer PT100 sensor with a specified accuracy of ± 0.3 °C. Accuracy as low as ± 0.05 °C can be reached with this same family of PT100s at a higher price [3]. The controller includes simultaneous cooling and heating with independent PWM that prevents overshooting while ensuring precise and accurate control within very narrow ranges at any reasonable temperature and does not risk damaging the heating or cooling elements. The controller has an internal clock that allows the user to set, and the controller to track, any temperature threshold at desired times of the day. It includes an output channel for grow light control that is paired with set day time temperatures. All output channels have independent 25A Solid-State-relays, in which a high amperage heater, AC, and grow lights can be directly connected without needing additional parts. Further, the control has an integrated Wi-Fi chip that transfers data via a local internet network to a web-application where the data are displayed in real time. This same functionally allows the controller to send warning emails for specific criteria (e.g., failing to reach a temperature threshold in certain time, which may indicate a failure in the system).

The controller consist of a PCB shield in which commercially available parts can be plugged to facilitate assembly and replacement. The full controller is enclosed in a 3-D printed casing to ensure safety standards for electrical insulation. The controller includes three female electrical sockets for connecting additional electrical devices if needed. The controller includes a liquid–crystal display (LCD) that displays the set threshold, the current temperature, and the time, date and phase period (dark or light). Controller settings are modified via a single rotary encoder. A rotary encoder, also called a shaft encoder, is an electro-mechanical device that converts the angular position or motion of a shaft or axle to analog or digital output signals; this functionality allows to loop over different variables of the controller but also set their values. All settings are stored in the EEPROM memory of the microcontroller such that they are not erased when the controller is unplugged. The control includes an internal 15 A push-in breaker, and connects directly to a 110 V electrical outlet. The full controller can be built for about $USD63, and be deployed in plant growing tents or nurseries and integrated with other controls (e.g., soil water content) to perform reliable, long-term experiments on plant performance.

### 2.1 Temperature control logic

**Fig. 2:**
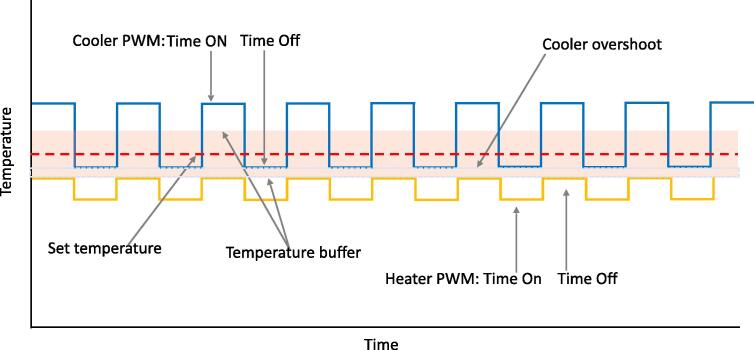
Temperature control logic. The goal of the controller is to keep air temperature within a buffer around a set temperature. This is done by activating an output channel with a heater when air temperature is below the lower buffer temperature or an output channel with a cooler when air temperature is above the upper buffer temperature.

The overall goal of the controller is to keep temperature within a buffer (Shadowed red area in Fig. 2 around a set temperature (dotted red line in Fig. 2. To achieve that, the controller has an output channel for a heating element (e.g., a room heater) that is activated when the air temperature is below the lower buffer temperature. The heating element is activated via PWM, in which the On or Off timing can be independently set. The time On and Off for the heating element should be fine-tuned to the given space and heater for optimal performance. The primary purpose of fine-tuning is to avoid overshooting temperature, which is case specific depending on the volume of the space to heat, insulation used and heating capacity of the heater. For instance, a 1,500 W heater can increase the temperature of a thermally insulated 128 ft^3^ chamber up to 10 °C is just below 30 sec. In this case, settings of 2 sec On and 10 sec Off for the heating element can slowly raise temperature to within the temperature buffer without overshooting. When the air temperature is above the upper temperature buffer, the controller activates a second channel where a cooling element (e.g., an AC) is connected. Cooling elements are not as fast at reducing temperature as heaters are at increasing temperature, so when cooling the risk of overshooting the lower temperature buffer is less likely; further the use of a compressor based AC prevents constant On and Off cycles. In the case of cooling, the output channel for the cooler is set to On any time the air temperature is above the upper buffer temperature and turned Off when the air temperature reaches the lower buffer temperature plus a set AC Overshoot. This ensures the air temperature never goes below the lower buffer temperature. For safety to the cooling element, a time Off setting is implemented, such that only after that time has lapse can the AC be set to On again.

### 2.2 Adjusting settings

**Fig. 3:**
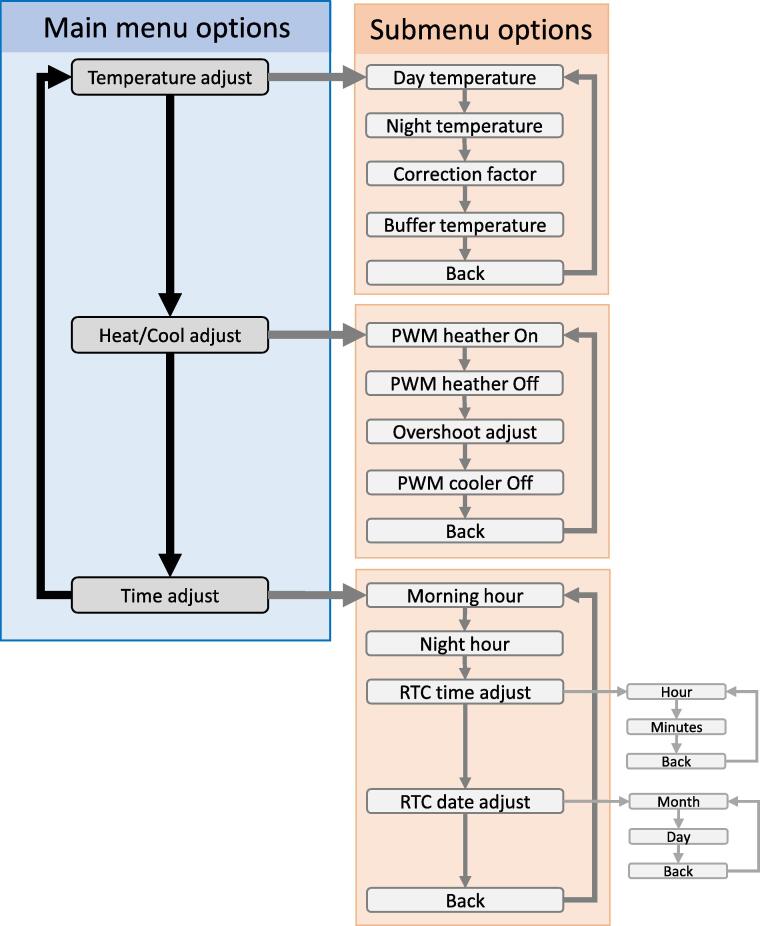
Settings of the temperature controller. To modify any specific setting of the controller, the encoder in the main body of the controller (See Fig. 1 is pressed, which will display a “Main menu of options” in the LCD. Pressing the encoder at any given menu option, will display a set of submenu options. Pressing on the submenu option will allow to modify the specific setting by turning the encoder right or left. After the given setting is at a desire value, the given value is stored in the EEPROM memory of the microcontroller by pressing the encoder. The controller will return to the main display after 30 sec if the encoder is not used after 30 s.

To adjust the controller settings, the encoder is pressed for 10 sec, which displays a menu of options in the LCD for the temperature threshold, the heating and cooling elements, and the time and date (Fig. 3. The encoder is turned left or right cycling through the different menu options; once the desire option is selected, the encoder is pressed to access sub-options for that specific setting (Fig. 3. The encoder is turned left or right cycling through the different sub-menu options, and pressed at the desire sub-menu option to modify the specific setting. The given setting can be increased or decreased by turning the encoder right or left, respectively. Once the given setting has been adjusted, the value will be stored in the EEPROM memory by pressing the encoder. The controller returns to control mode after the encoder is inactive for 30 sec.

## 3 Design files

### Design files summary

**Table:** 

Design file name	File type	Open source license	Location of the file
IPSTempNANO	Arduino code	CC BY 4.0	https://doi.org/10.17605/OSF.IO/J7ZKV
IPSTempWIFI	Arduino code	CC BY 4.0	https://doi.org/10.17605/OSF.IO/J7ZKV
IPSCircuit	7z	CC BY 4.0	https://doi.org/10.17605/OSF.IO/J7ZKV
ElectricControllerBox	7z	CC BY 4.0	https://doi.org/10.17605/OSF.IO/J7ZKV

**IPSTempNANO:** This file provides the Arduino code to operate the controller. The code is provided with comments on the margins to explain each line of command.

**IPSTempWIFI:** This file provides the Arduino code to operate the WIFI microcontroller. The code is provided with comments on the margins to explain each line of command. A video describing the steps for the web interface of the controller is provided here.

**IPSCircuit:** This folder contains four files needed to print the PCB shield for the controller. The shield adjusts to an Arduino Nano and allows to plug in off-the-shelf parts for easy assembly. Files can be opened in the circuit board design software, Eagle. This folder also includes the schematics of the electrical circuit. Details about the free installation of Eagle is available here.

**ElectricControllerBox:** This folder contains two SolidWorks files for 3D printing the controller’s case (the box and the cover). The case is for electrical insulation and to allow the placement of the electrical sockets for the controller output channels and three electrical female sockets as additional power outlets.

## 4 Bill of materials

The estimated total cost for materials is about $USD63. And other than the PCB and the 3D print, all other parts can be purchased off-the-shelf.

Bill of materials

**Table:** 

Designator	Component	Number	Cost per unit $USD	Total cost $USD	Source of materials	Material type
IPSCircuit	Arduino Nano	1	$4.00	$4.0	URL	*Microcontroller*
IPSCircuit	0.96 Inch OLED Display Module	1	$1.60	$1.6	URL	LCD
IPSCircuit	25Amp Solid State Relay	3	$8.00	$24.0	URL	Electric
IPSCircuit	PT100 sensor	1	$1.00	$1.0	URL	Sensor
IPSCircuit	MAX31865 Sensor Amplifier	1	$4.20	$4.2	URL	Electric
IPSCircuit	Female Sockets NEMA Panel Mount	6	$1.00	$6.0	URL	Electric
IPSCircuit	DC Push Button Circuit Breaker	1	$7.00	$7.0	URL	Electric
IPSCircuit	2A AC/DC adapter	1	$4.00	$2.0	URL	Electric
IPSCircuit	ESP-01S ESP8266 WIFI board	1	$2.00	$2.0	URL	*Microcontroller*
IPSCircuit	AC 110v to 5v DC converser	1	$3.20	$3.2	URL	Electric
IPSCircuit	Encoder	1	$0.50	$0.5	URL	Electric
IPSCircuit	DS3231 Real time Clock Memory	1	$1.90	$1.9	URL	Electric
IPSCircuit	PCB	1	$2.00	$2.0		Hardware
ControllerBox	3D print	1	$4.00	$4.0		Hardware
			**Total→**	**$63.4**		

## 5 Build instructions

The construction of the controller is divided into three sections. First, the soldering of the terminals in the PCB shield in which the electrical parts are plugged into. Second, assembling the electrical circuit with the female sockets for the controller’s three output channels (i.e., heating, cooling and light) and the three additional female sockets for extra electrical outlets. Third, setting up the web-interface for data storage and display. Each of these three steps are documented in detail in supplement files 1 to 4 (https://doi.org/10.17605/OSF.IO/J7ZKV).

## 6 Operation instructions

To operate the controller, connect a heater in the output heating channel, and a cooler (e.g., AC) in the output cooling channel. Place the temperature probe inside the space in which temperature is to be controlled. Connect the controller to a 110 V electrical outlet and the controller is ready to work based on the default settings stored in the microcontroller EEPROM memory.

## 7 Validation and characterization

### 7.1 Accuracy

To study temperature control accuracy, we distinguish between i) sensor accuracy, which is the ability of the controller to accurately measure the actual temperature of the chamber, and ii) controller accuracy, which is the ability of the controller to maintain the temperature at the set point. Sensor accuracy was the absolute difference in air temperature as measured by the controller’s PT 100 sensor and a high accuracy RTD sensor. Controller accuracy was the absolute difference between the set point temperature and the measured air temperature by a high accuracy RTD sensor.

**Fig. 4:**
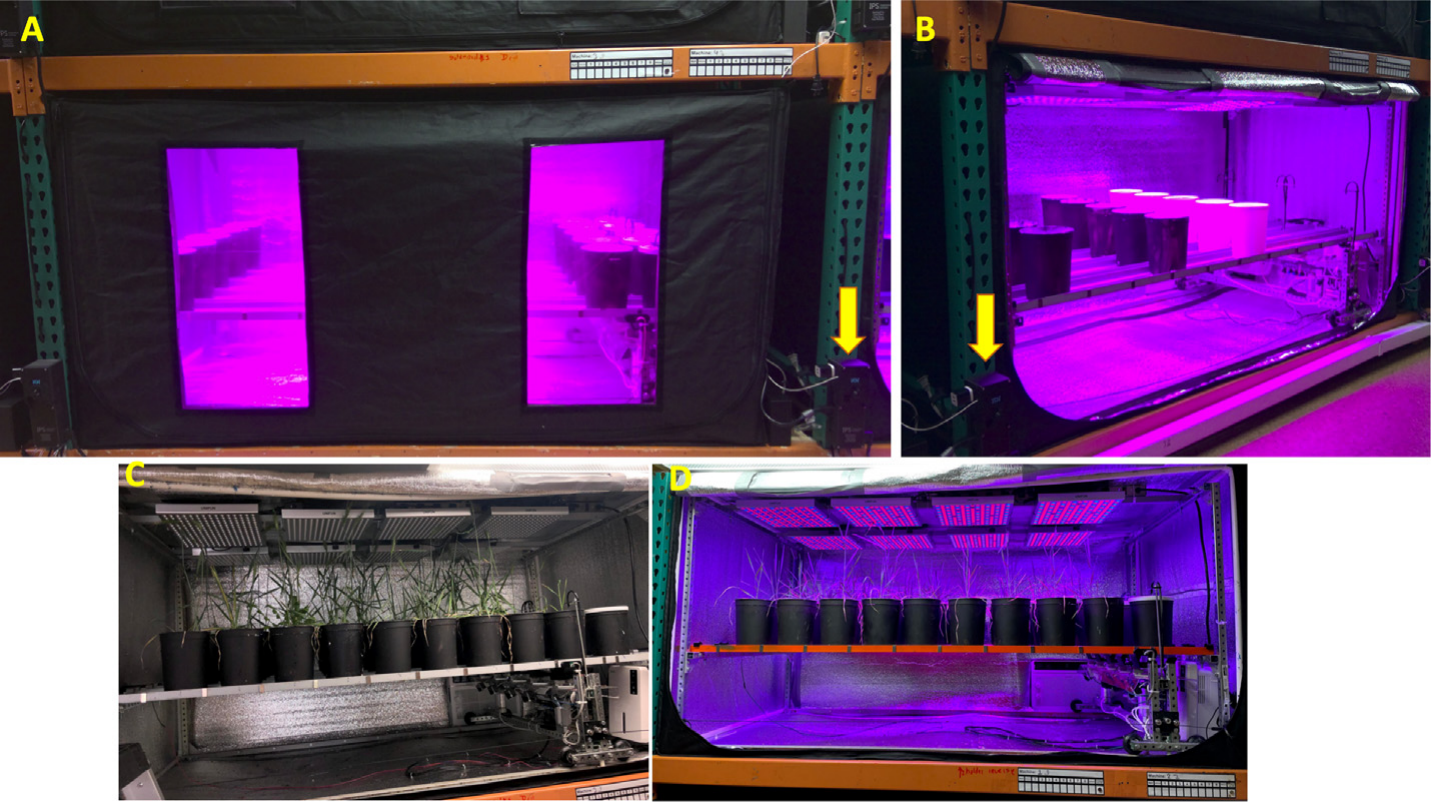
Experimental tent/chamber for plants. A. Chamber closed. B. Chamber opened. The position of the controller is indicated with the yellow arrows. C-D chamber with wheat plants. (For interpretation of the references to colour in this figure legend, the reader is referred to the web version of this article.)

To check both types of accuracy, a prototype controller was used to control the temperature in a 4 × 4 × 8 ft thermally insulated tent (i.e. chamber, Fig. 4. We ran experiments at six different set point temperatures (15, 20, 25, 30, 35, and 40 °C) and repeated each experiment twice. In all experiments, the programmed buffer zone was ± 0.3 °C. Temperature measurements from the controller’s internal temperature sensor (PT100) and an independent high accuracy digital thermometer were recorded every 10 sec for 5 min. The independent digital thermometer was a Market Lab Traceable High-Accuracy RTD General Purpose Digital Thermometer that is accurate to ± 0.1 °C.

#### 7.1.1 Sensor accuracy

**Fig. 5:**
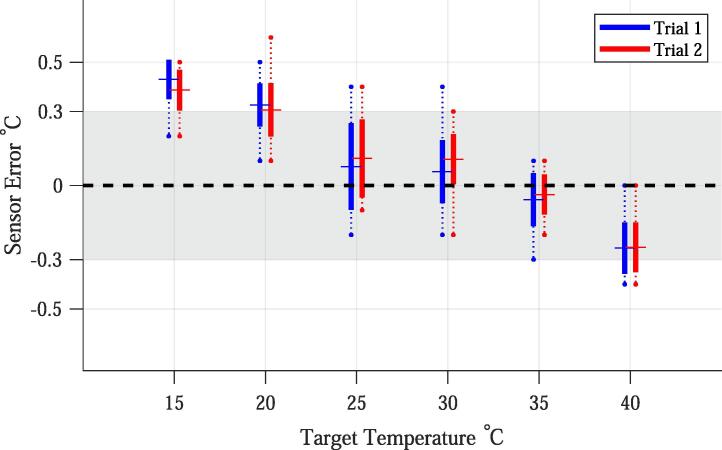
Sensor accuracy; defined as the difference between the temperature measured with a High-Accuracy RTD Digital Thermometer and the temperature measured with the controller’s PT100 sensor: ($T_{\mathrm{RTD}}-T_{PT100}$). For each target temperature, the total range (dotted line), first standard deviation (solid line), and mean (crossing line) are plotted. Also displayed is the +/-0.3 °C buffer zone (grey shadowed area). Ambient temperature was commonly between 22 °C and 24 °C.

The overall average absolute temperature difference between the controller’s PT100 sensor and the RTD sensor (i.e. mean[abs(T_PT100-T_RTD)]) was 0.2 °C with a standard deviation of 0.2 °C. The controller’s PT100 sensor measurements were lower than the RTD measurements at low temperatures and higher than the RTD measurements at higher temperatures (Fig. 5.

#### 7.1.2 Controller accuracy

**Fig. 6:**
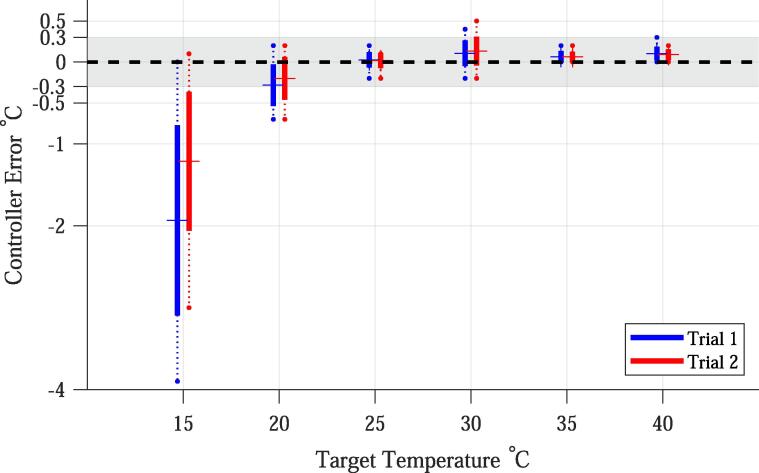
Controller accuracy; defined as the difference between the set point/target temperature and the temperature measured with an external high accuracy thermometer ($T_{\mathrm{Target}}-T_{\mathrm{RTD}}$). For each target temperature, the total range (dotted line), first standard deviation (solid line), and mean (crossing line) are plotted. Also displayed is the +/-0.3 °C buffer zone (grey shadowed area). Ambient temperature was commonly between 22 °C and 24 °C. See body of paper for explanation of the larger controller accuracy error at low temperatures.

The overall average absolute temperature difference between the set point temperature and the measured temperature (i.e. mean[abs(T_set-T_RTD)]) was 0.3 °C with a standard deviation of 0.6 °C (Fig. 6. However, the controller accuracy error was larger when controlling below ambient temperatures, and within the defined buffer when the temperature controlled was at or above ambient temperature; we review this pattern next.

##### 7.1.2.1 Below ambient temperature control

As seen in Fig. 6, the controller accuracy decreases with decreasing temperature. At the lowest temperature (about 10 °C below ambient temperature), the controller accuracy was −1.4 °C with a standard deviation of 1.0 °C. The negative average indicates the actual temperature was on average higher than the desired set point. The larger error at lower temperatures was due to the inability of the AC cooling system utilized in the experiment to quickly compensate for the chamber heat loss given the set PWM. We found that the chamber tested, when set at 10 °C below ambient temperature, exhibits a heat loss that results in a temperature increase of ∼ 1 °C/min. In turn, the AC used to cool down the chamber required a 60 sec Off-state before it could be set to On again. Turning the AC On any sooner prevented the compressor from starting since it was still under the pressure load from the previous On phase. This failure to control below ambient temperatures is not necessarily an error of the controller itself, but a limitation of the cooling element. With this in mind, increasing accuracy at low temperatures could be reached with complementary ACs, thermoelectric coolers that can tolerate faster On and Off-phases, or better thermal insulation of the chambers to reduce the rate of heat loss.

##### 7.1.2.2 Equal to or above ambient temperature control

As seen in Fig. 6, for set point temperatures above ambient temperatures, the controller maintained temperature well within the buffer zone. The average temperature difference between the set point temperature and the measured temperature for all experiments with set points above or nearly equal to ambient was 0.1 °C with a standard deviation of 0.1 °C (Fig. 6. The high controller accuracy at ambient or higher temperatures is due to the high heating power of the elements used and the capacity for them to remain functional while quickly being switched On and Off (i.e., they can quickly replenish heat loses of the enclosed space).

### 7.2 Controller long term reliability

To test the long-term reliability of the controller, we run a long-term experiment to quantify biomass variation in wheat along a temperature gradient. For this experiment, we set five 128 ft^3^ tents –or chambers (Fig. 4 at five different temperatures to be kept within a buffer of 1 °C. The chambers were complemented with a recently automated machine to control soil water content [4]; we used five different soil water content treatments equally spaced between 10% and 90%. Each treatment of temperature and soil water content was replicated in eight plants. For this experiment, wheat seeds were placed in moist paper until germination, then transplanted into 1 Gal pots, and placed randomly into the chambers. Seedlings were left to acclimatize for one week in the chambers with a grow light cycle of 12 h light and 12 h dark. After the acclimation week, the treatments of water and temperature were activated for six weeks until plants reached maturity. Adult wheat plants were collected at the end of the experiment to measure their total dry biomass.

**Fig. 7:**
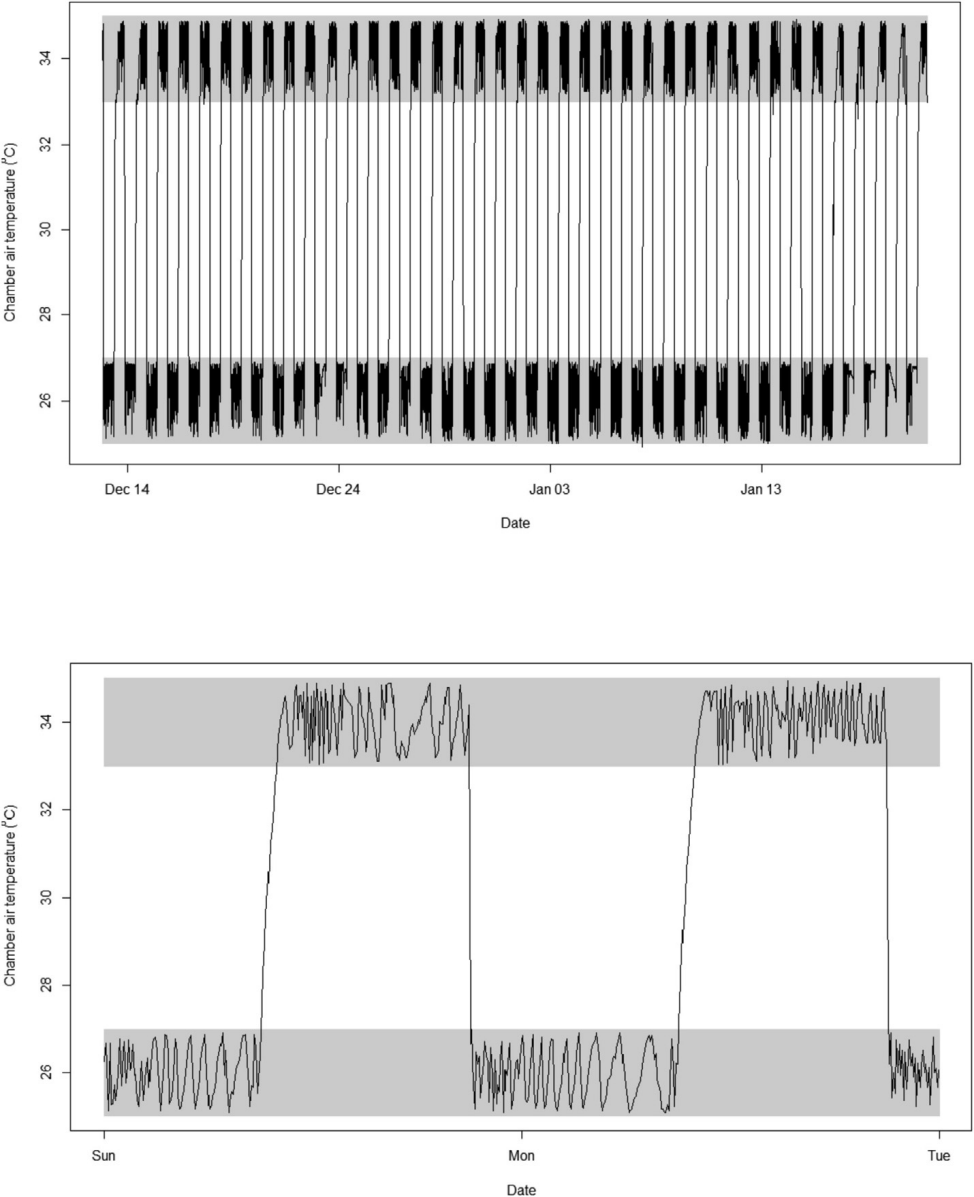
Repeatability of a diurnal temperature cycle over a long-term plant experiment. Displayed here are six minutes temperature readings in a long term experiment in which wheat plants were grown from seedling to maturity. In this case example, day temperature was set at 34 °C and night temperature at 26 °C, with a temperature buffer of 1 °C. The gray area indicates the buffer zones.

**Fig. 8:**
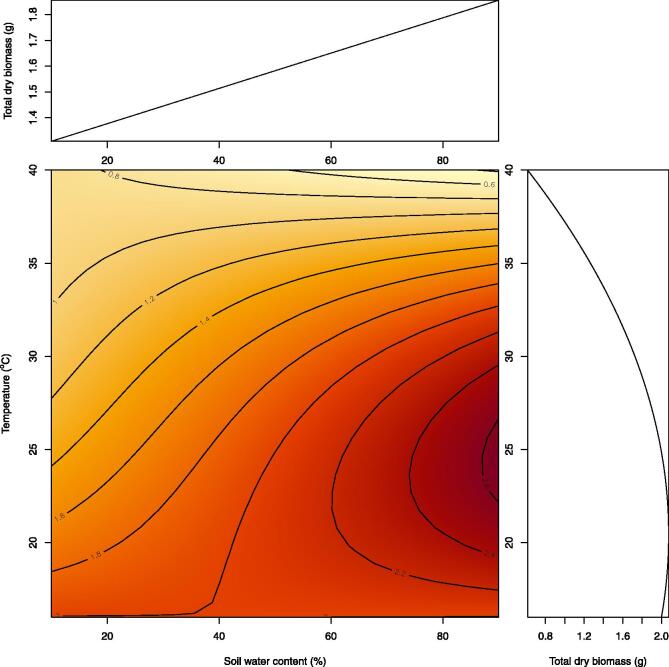
Effect of temperature and soil water content on wheat biomass. Displayed here are the biomass responses of wheat plants grown under a fully factorial experiment of soil water content and temperature over the full range of conditions relevant to this species.

**Fig. 9:**
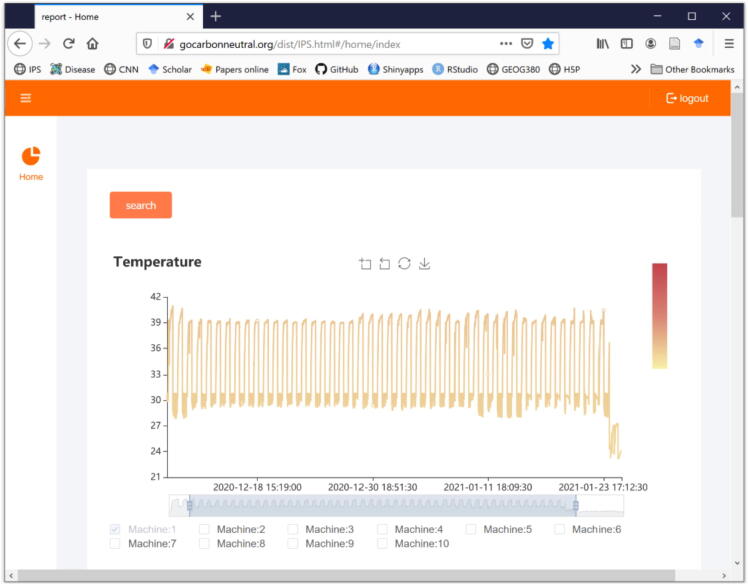
Web-display of real-time temperature data from the temperature controller. Data collected by the controller is transmitted via a local WIFI network to a web-page, in which data are visualized in real time. The web interface allows to display data from up to ten controllers simultaneously. Displayed here are the data for one of the chambers used in the experiment outlined in the body of the paper.

Over the duration of the experiment, the temperature controller maintained a highly repeatable dark/light temperature cycle within the set buffer (Fig. 7. The effects of temperature and water were significant in wheat biomass at p < 0.0001 (Fig. 8. Expectedly, the effect of temperature on biomass was significantly non-linear with biomass peaking at an optimum temperature around 25 °C, and declining towards colder and hotter temperatures (Fig. 8. These results indicate the long-term repeatability and reliability of the temperature controller for experiments on plant grow. The web-display of the temperature data over the duration of the experiment, for one of the chambers, is shown in Fig. 9.

## 8 Discussion

Studying the effect of temperature in biological systems has long been of interest to numerous scientific and applied endeavors, but has gained particular interest in the field of plant biology with respect to the potential effect of greenhouse gases on the Earth’s temperature and how this could translate to impacts on plant biomass production. Insight into these questions are of interest to climate modelers over the role of plants on carbon sequestration and temperature regulation [5], and to plant ecologists over the need to select heat tolerant plants to feed humanity [6]. Unfortunately, insights into these questions have been considerably hampered by the economic unfeasibility of implementing available technologies to carry out the needed large-scale experiments. Assessing the impacts of climatic variables on plants calls for large factorial experiments. Each combination of climatic variables, plus enough replication, commonly requires independent environmentally controlled chambers that quickly skyrockets the economic cost of such experiments [e.g., climate controlled chambers for plants can be upwards of $10,000 a piece; e.g., [2]]. Here we report an accurate, and simple to operate temperature controller that can be easily replicated and used in small plant grow tents to successfully carry out plant experiments at relatively low cost. Further, the controller can be integrated with an also available controller for soil water content [4], which allows for more complex factorial experiments over the broad ranges of temperature and water variability relevant to plants. We hope this reliable and affordable capacity to carry out factorial experiments will allow for more studies and accelerate our understanding of the impacts of climatic changes on plant performance.

## Declaration of Competing Interest

The authors declare that they have no known competing financial interests or personal relationships that could have appeared to influence the work reported in this paper.

## References

[1] Wavelength Electronics, TC5 LAB Series 5 A Temperature Control Instrument, https://www.teamwavelength.com/product/tc5-lab-series-5a-temperature-control-instrument?gclid=Cj0KCQjw0caCBhCIARIsAGAfuMwSfvPiEVQe-FCOIWPdyqpEFkrJzOXFoW_wNeAuFwztgYQ_z7YwSskaAoDGEALw_wcB.

[2] Thermo Fisher Scientific, PR505750L - Thermo Scientific PR505750L Refrigerated Incubator with Day/Night Cycle, https://www.neobits.com/thermo_fisher_scientific_pr505750l_thermo_p15438476.html?atc=gbp&gclid=CjwKCAjw9MuCBhBUEiwAbDZ-7riq-86gISoM9-50T5h4XVbDzae8-eslIPpLa6MsYXu6EOtBjsbd6RoCdhkQAvD_BwE.

[3] Testo, Highly accurate Pt100 immersion/penetration probe, https://www.testo.com/en-US/highly-accurate-pt100-immersion/penetration-probe/p/0614-0235.

[4] Takara G., Zachary Trimble A., Arata R., Brown S., Jaime Gonzalez H., Mora C. (2021). An inexpensive robotic gantry to screen and control soil moisture for plant experiments. Hardware X.

[5] Mora C., Caldwell I.R., Caldwell J.M., Fisher M.R., Genco B.M., Running S.W. (2015). Suitable days for plant growth disappear under projected climate change: Potential human and biotic vulnerability. PLoS Biol..

[6] Tubiello F.N., Soussana J.-F., Howden S.M. (2007). Crop and pasture response to climate change. Proc. Natl. Acad. Sci..

